# An MRI-based method to register patient-specific wall shear stress data to histology

**DOI:** 10.1371/journal.pone.0217271

**Published:** 2019-06-06

**Authors:** A. M. Moerman, K. Dilba, S. Korteland, D. H. J. Poot, S. Klein, A. van der Lugt, E. V. Rouwet, K. van Gaalen, J. J. Wentzel, A. F. W. van der Steen, F. J. H. Gijsen, K. Van der Heiden

**Affiliations:** 1 Department of Biomedical Engineering, Erasmus MC, Rotterdam, The Netherlands; 2 Department of Radiology and Nuclear Medicine, Erasmus MC, Rotterdam, The Netherlands; 3 Department of Medical Informatics, Erasmus MC, Rotterdam, The Netherlands; 4 Department of Surgery, Erasmus MC, Rotterdam, The Netherlands; Worcester Polytechnic Institute, UNITED STATES

## Abstract

Wall shear stress (WSS), the frictional force exerted on endothelial cells by blood flow, is hypothesised to influence atherosclerotic plaque growth and composition. We developed a methodology for image registration of MR and histology images of advanced human carotid plaques and corresponding WSS data, obtained by MRI and computational fluid dynamics.

The image registration method requires four types of input images, *in vivo* MRI, *ex vivo* MRI, photographs of transversally sectioned plaque tissue and histology images. These images are transformed to a shared 3D image domain by applying a combination of rigid and non-rigid registration algorithms. Transformation matrices obtained from registration of these images are used to transform subject-specific WSS data to the shared 3D image domain as well. WSS values originating from the 3D WSS map are visualised in 2D on the corresponding lumen locations in the histological sections and divided into eight radial segments. In each radial segment, the correlation between WSS values and plaque composition based on histological parameters can be assessed. The registration method was successfully applied to two carotid endarterectomy specimens. The resulting matched contours from the imaging modalities had Hausdorff distances between 0.57 and 0.70 mm, which is in the order of magnitude of the *in vivo* MRI resolution. We simulated the effect of a mismatch in the rigid registration of imaging modalities on WSS results by relocating the WSS data with respect to the stack of histology images. A 0.6 mm relocation altered the mean WSS values projected on radial bins on average by 0.59 Pa, compared to the output of original registration. This mismatch of one image slice did not change the correlation between WSS and plaque thickness. In conclusion, we created a method to investigate correlations between WSS and plaque composition.

## Introduction

Atherosclerosis is a progressive vascular disease, characterised by the accumulation of lipids and inflammatory cells in the vessel wall, which results in plaque formation. A subset of atherosclerotic plaques is prone to rupture [1–3]. A rupture-prone, vulnerable plaque differs compositionally from a stable plaque, and is characterised by a large lipid core covered by a thin fibrous cap, inflammatory cell infiltration and/or intraplaque haemorrhage. In the event of rupture, plaque- and thrombus material may embolise into the distally located vessel bed. Depending on the anatomical location of the plaque, rupture might lead to stroke or acute myocardial infarction. Unravelling the mechanisms behind plaque destabilisation, leading to a rupture-prone plaque, is thus of high importance.

Wall shear stress (WSS) is the blood-exerted frictional force on the vessel wall. Low and/or oscillatory WSS is an established factor in atherosclerosis initiation, due to activation of pro-inflammatory pathways in the endothelium. The pro-inflammatory environment favours oxidation and retention of lipoproteins inside the vessel wall, aggravating inflammation and resulting in atherosclerotic plaque formation [4–6].

In case of advanced lumen-intruding plaques, the influence of WSS on the human plaque composition and thus vulnerability is still a subject of debate. Several invasive imaging studies have assessed plaque size and corresponding WSS levels [7–9] while other studies included surrogate markers of plaque vulnerability, such as plaque burden [10–14], intraplaque heamorrhage [15, 16], inflammation [17], lipid core size [18] or plaque stiffness [19]. However, none of these studies were able to fully characterise plaque vulnerability as histology is regarded as the gold standard for assessment of local plaque vulnerability. Thus, our research question requires co-registration of 2D histology information with MR imaging and its derived WSS data. Co-registration of imaging modalities is challenging, as the registration method has to account for tissue reorientation and deformation occurring due to multimodal imaging and tissue processing. Different approaches of registering histology sections and 3D medical imaging such as MRI have been proposed, varying from slice-to-slice approaches, slice-to-volume approaches, volume-based approaches and hybrid methods. Also, extra imaging modalities, such as *ex vivo* tissue scans and/or blockface photographs, have been added to registration frameworks to refine registrations and account for tissue processing artefacts [20]. Multiple methods for co-registration have been described in an extensive review [20].

To assess the relation between WSS and plaque composition from histology, we based our image registration method on a previously designed tool [21] to map 2D histological cross-sections of a human carotid plaque to the 3D *in vivo* artery geometry. [21–23]. In our method, the registration of histology and *in vivo* MRI/WSS data was aided by an additional *ex vivo* MR scan and block photographs (en face) of sliced tissue. The novelty of the method presented here, lies in the use of subject-specific geometries and flow data obtained by *in vivo* MR imaging, making the image registration framework fully based upon MRI data.

In this paper, the new image registration method is described and tested on two human carotid plaques. Moreover, we simulated the effect of a registration mismatch between imaging modalities on WSS results to evaluate the impact of a potential mismatch on WSS and correlations between WSS and plaque thickness.

## Materials and methods

### Plaque MR imaging

#### In vivo MR imaging

Two patients scheduled for elective carotid endarterectomy for recent stroke or transient ischemic attack, underwent an MRI scan one day prior to surgery. The carotid plaques were imaged *in vivo* in order to visualise lumen and outer wall geometry and measure blood flow velocity. Patients were scanned in a 3.0 T MRI scanner (General Electric (GE) Healthcare, Milwaukee, USA) using a four-channel phased-array coil with an angulated setup (Machnet B.V., Roden, The Netherlands). Lumen and plaque geometry were imaged using a black blood 3D fast spin echo (3D-BB-FSE) sequence with variable flip angles (TR/TE: 1000/16 ms, FOV: 15 cm, slice thickness: 0.8 mm, matrix: 160x160, number of excitations 1, scan time: 190 s). The MRI scan was resampled to a resolution of 0.4 x 0.4 x 0.6 mm. Blood flow velocity was measured at 2 locations, approximately 20 mm below and ~20 mm above the carotid bifurcation, using 3D phase-contrast MRI (TR/TE: 5/3 ms, FOV: 15 cm, slice thickness: 4.0 mm, matrix: 160x160, scan time: ~3 min, VENC: 70 cm/s). Written informed consent was obtained. This study was approved by the Medical Ethical Committee of Erasmus MC.

#### Plaque tissue collection and ex vivo MR imaging

Carotid plaque specimens, hereafter referred to as ‘CEA1’ and ‘CEA2’, were collected within 30 minutes after surgical resection. Plaque tissue was resected with a specialised technique, resulting in tissue specimens with intact lumen and plaque morphology [24]. Tissue was rinsed with phosphate-buffered saline (PBS), snap frozen in liquid nitrogen and stored at -80°C until further processing.

*Ex vivo* T2w fast recovery FSE (frFSE)MRI scans (TR/TE: 2500/66 ms, in-plane resolution: 0.1 x 0.1 mm, slice thickness: 0.5 mm, matrix: 256x256, scan time: ~20 min, number of slices: 66) of the excised CEAs provided necessary images of the plaque to link histology and *in vivo* MRI images in the registration procedure. *Ex vivo* MR imaging was performed on 4% formaldehyde-fixed plaque specimens, immersed in PBS, with a 7.0T MRI scanner (7.0T Discovery MR901, GE Healthcare, Milwaukee, USA).

### Specimen processing, en face photos and analysis

**Fig 1:**
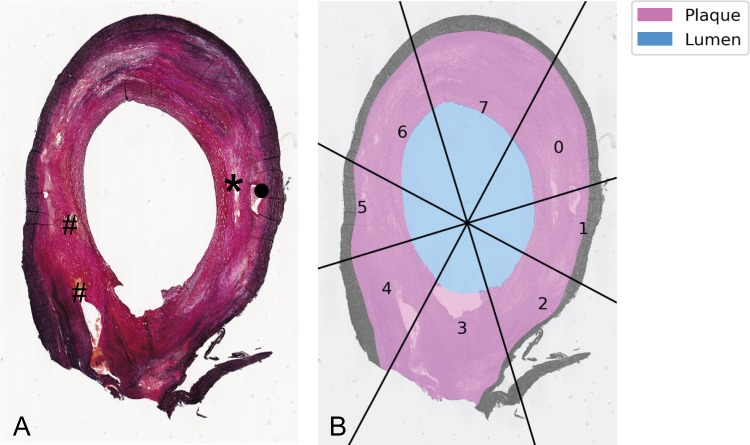
Result of manual segmentation of lumen and intima areas and definition of radial bins. **(**A) Image of an histology section (Miller's Elastic Stain) of caudal side of an endarterectomy specimen. Plaque components are visualised: * necrotic core, • calcium, # heamorrhage. (B) Segmentation of intima and lumen area and radial bins. Based on the centerline, eight radial bins were defined. Mean WSS per radial bin was calculated.

Formaldehyde-fixed CEA tissue was decalcified in a solution of 10% ethylenediaminetetraacetic acid (EDTA) in demineralised water for 14 days, washed in PBS and cut in 1 mm consecutive transverse sections. The proximal side of each transverse section was photographed (IXUS 60, Canon, Tokyo, Japan). Photos, hereafter referred to as 'en face photos', were taken from a fixed point perpendicular to the tissue section. The tissue section was further processed and embedded in paraffin. The en face photo contained landmarks to enable the registration of this photo to the en face photo of the adjacent transverse section, as well as a measuring grid to calculate the image resolution. The paraffin blocks were sectioned into 5 μm sections, which were stained histochemically (Hematoxilyn-Eosin stain and Miller's Elastic stain). Lumen and intima were segmented on the Miller's Elastic stain (Fig 1), using an in-house developed software tool (Mevislab 2.7.1, MeVis Medical Solutions, Bremen, Germany). Size of plaque components was expressed in mm^2^.

### Computational fluid dynamics

Lumen contours were segmented from the MRI 3D-BB-FSE scan using ITK-SNAP software [25]. A volume mesh of approximately 4 million tetrahedral and prism elements was generated using ICEM (ANSYS ICEM, 17.1, ANSYS, Pennsylvania, USA). The patient-specific time-dependent blood velocity profile in the common carotid artery (CCA) was derived from the phase-contrast MRI scan using MATLAB (MATLAB R2015b, Mathworks Inc., Natick (MA), USA) and applied as inlet boundary condition [26]. As outlet boundary condition we assumed the outflow ratio of the internal carotid artery (ICA) and external carotid artery (ECA) to be 50:50, corresponding to moderately stenosed carotid bifurcations [27]. According to previously defined protocols, blood density was set to 1060 kg/m^3^ and non-Newtonian fluid behaviour was mimicked by the Carreau-Yasuda model [26]. The Navier-Stokes equations were solved and time-dependent wall shear stress (WSS) was computed using Fluent software (ANSYS Fluent, 17.1, ANSYS, Pennsylvania USA) over the length of two heart cycles with a time step of 0.004 seconds. The results of the second heart cycle were used for analysis to account for entrance effects.

### Image registration

In order to register 2D histology images to the 3D shear stress distribution, we adapted an image registration tool that was developed to register *in vivo* CT images to histology [21].

**Fig 2:**
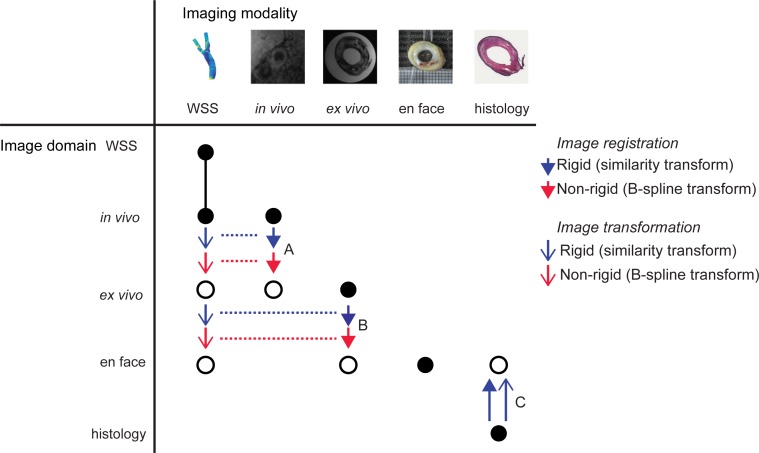
Image registration overview. Black dots represent the original image domains of the different imaging modalities. White dots represent the image domain where the corresponding imaging modality is mapped to using rigid (blue arrows) and non-rigid (red arrows) transformations. Image sets are brought to a mutual image domain, the en face photos. Step A represents the registration of *in vivo* MRI to *ex vivo* MRI. Step B represents the registration of *ex vivo* MRI to en face photos. The coordinate transformations obtained in registration step A and B can also be used to map the WSS data to the image domain of the en face photos. Step C represents the registration and transformation of histology images to the en face image domain.

We aimed at mapping histology images, en face photos, *ex vivo* MRI and *in vivo* MRI, as well as the WSS data to a shared 3D image domain. We chose the image domain of the en face photos as the shared domain. To that aim, MRI scans and WSS data were resampled and transformed to the en face image domain by the series of registration steps explained below and shown in Fig 2. The z-resolution of en face and histology was equal, since each en face photo had a corresponding histology section. Thus by choice of the shared en face image domain, interpolation of histology images in z-direction could be avoided and only in-plane registrations were necessary to transform histology to the shared image domain. Coordinate transformations that describe mappings of the different imaging modalities were obtained in a series of registration steps. An overview of the registration procedure is shown in Fig 2.

A 3D reconstruction of the excised vessel was created by stacking the en face photos of adjacent transverse sections, using point-based rigid registration based on applied landmarks. In this way, we obtained a 3D stack of en face photos. Subsequently, *in vivo* MRI, *ex vivo* MRI and histology images were mapped to the 3D stack of en face photos via a series of registration steps. We started with rigid registration of *in vivo* MR images to *ex vivo* MR images, by determining corresponding points on the first slice cranial to the bifurcation in both image sets (Fig 2, step A, blue arrow). This was the first MR slice in which both the internal and the external carotid artery were visible. Based on these points and using a similarity transformation, i.e. rotation and isotropic scaling, the *in vivo* MR images were transformed and resampled to the *ex vivo* image domain using the Elastix toolbox [28]. We assumed the deformation of the plaque in longitudinal direction after surgical resection to be minimal, as the tissue was relatively stiff and was resected in intact shape. After rigid registration, an additional B-spline deformation model was applied to the transformed and resampled *in vivo* MR images to improve the mapping to the *ex vivo* MR images [28]. To that aim, contours of the lumen and outer wall were drawn in both image sets. A non-rigid B-spline transformation (metric: advanced mean squares, optimisation: adaptive stochastic gradient descent [28]) was applied to match the *in vivo* contour sets to the *ex vivo* contours (Fig 2, step A, red arrow). The coordinate transformation resulting from this non-rigid registration step was applied to the *in vivo* MR images that were already rigidly registered to the *ex vivo* images. Thus, with the aid of a similarity and a B-spline transform, the *in vivo* MR images were mapped to the *ex vivo* image domain The *ex vivo* MR images were registered to the 3D stack of en face photos (Fig 2, step B) using a similar rigid and non-rigid registration procedure. Based on anatomical landmarks, histological sections were registered and transformed to their corresponding section in the 3D stack of en face photos using a similarity transformation (Fig 2, step C). This resulted in a 3D stack of histology images. Taken together, this set of registration steps provided us with the necessary coordinate transformations to map the WSS data and the *in vivo* MR images to the 3D stack of en face photos as well.

### Data processing, selection and analysis

**Fig 3:**
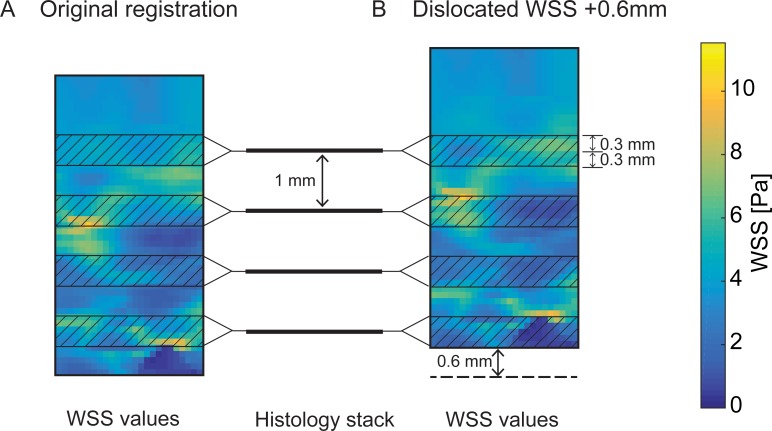
**Projection of WSS data on histology (A) and simulation of image registration mismatch (B).** A illustrates the relation between WSS data, which is continuous in z-direction, and the 3D reconstructed stack of histology sections, which are spaced 1mm in z-direction. For analysis of the correlation between WSS and histology, WSS data needed to be averaged in z-direction and mapped and correlated to the nearest histology image. WSS data was averaged in z-direction over a region of -0.3 mm to +0.3 mm with respect to the z-location of the histological section. B illustrates how the WSS data was relocated with respect to the histology stack to simulate a mismatch in registration.

For data analysis, WSS values originating from the 3D WSS map were visualised in 2D onto the corresponding lumen locations in the histological sections using nearest-neighbour interpolation. Therefore, WSS values were averaged in axial direction over a region of -0.3 mm to +0.3 mm with respect to the z-location of the histological section (Fig 3A). This axial length corresponded to the axial resolution of the *in vivo* MRI scan. In-plane, the cross-section was subdivided into 8 radial segments and the WSS values were averaged [29]. The centerpoint that served as origin for the radial segmentation, was obtained from the centerline of the transformed 3D WSS map. This centerline was obtained using the centerline algorithm in VMTK software [30] which makes use of the Voronoi diagram of the vessel model. The centerline calculation is based on the radii of maximally inscribed spheres along the path of the Voronoi diagram [31]. An example of a histological image with manually segmented areas and distribution of radial bins is shown in Fig 1.

Radial bins were eliminated from the dataset on the basis of three types of errors: 1) presence of processing artefacts in histology, 2) mismatch between histology and en face photos due to inhomogeneous shrinkage or strain in the tissue that could not be accounted for in the registration procedure, and 3) mismatch in registration between *in vivo* MR images and en face photos. The presence of all error types was visually assessed by three independent observers. Radial bins were excluded based on consensus. The effect of the exclusion of type 2 and type 3 error-containing bins was investigated with the following metrics: 1) by calculating the Dice Similarity Coefficient (DSC) between lumen segmentations in the en face photos and histology images (type 2 error, DSC_type2) and in the en face photos and transformed *in vivo* images (type 3 error, DSC_type3) and 2) by calculating the Hausdorff distance (HD) between the edges of the lumen segmentations in the en face photos and histology images (HD_type 2) and in the en face photos and in the transformed *in vivo* images (HD_type3). For each radial segment, mean, maximum and minimum WSS (Pa) and average plaque thickness (mm) were calculated. Plaque thickness was defined as the mean shortest distance between the lumen border and the lamina media border in histology images.

### Simulation of image registration mismatch

This procedure used a combination of imaging modalities and multiple registration steps to enable reliable registration of WSS data to histology. We investigated the effect of a potential registration mismatch in the order of magnitude of the *in vivo* MR resolution on mapped WSS values. To that aim, we relocated the WSS data with respect to the stack of histology slices. We simulated mismatches in rigid registration of a complete axial *in vivo* MRI slice, i.e. a relocation of WSS data by -0.6 mm or +0.6 mm, and a mismatch of half the axial *in vivo* MRI resolution, i.e. -0.3 mm and +0.3 mm (Fig 3). We subsequently analysed the resulting change in WSS value projected on each radial histology bin. The effect of registration mismatches on the possible correlation between WSS and plaque thickness was also investigated by plotting, for each radial bin, the mean, minimum and maximum WSS against the average plaque thickness and calculating the Pearson correlation coefficient R.

### Statistical analysis

WSS values projected on radial bins, after different relocation distances of the WSS stack, were compared to the original WSS-histology registration using a Wilcoxon signed rank test. For each relocation case, the correlation between WSS and plaque thickness per radial bin was calculated (MATLAB R2015b, Mathworks Inc., Natick (MA), USA) at a 0.05 significance level.

## Results

### Plaque imaging, specimen processing and image registration

**Fig 4:**
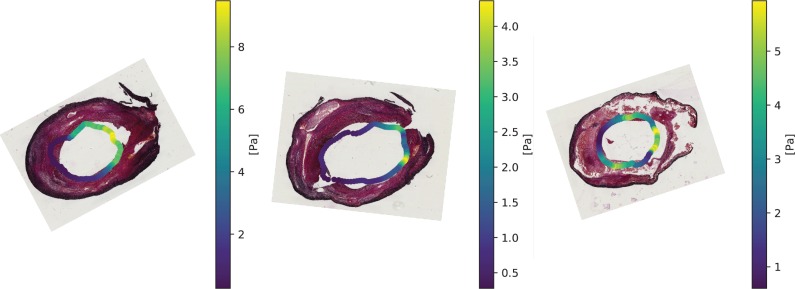
Histology images with WSS data projected onto the lumen. This figure illustrates the mapping of histology sections and WSS data to the shared image domain by the series of registration steps. When brought into the same image domain, WSS data, averaged in axial direction, can be projected onto the lumen of the corresponding histology section.

12 sets of WSS-histology images, representing 12 axial locations, were included for CEA1 and 11 sets of images for CEA2. In Fig 4 shows some examples of histology slices with the transformed WSS data visualised on the lumen.

### Data processing, selection and analysis

We segmented the lumen and intima contours on all histological sections included in the analysis. Using the centerline as origin, eight radial bins were projected onto a histological section. This resulted in (12+11)*8 = 184 radial bins in total. Per radial bin, we checked whether histological processing errors (type 1) or registration errors (type 2 and 3) were present. In Table 1 the number of excluded radial segments after image registration on the basis of different errors is summarised for CEA1 and CEA2. Note that some bins present with multiple type of errors.

**Table 1:** Exclusion of radial bins on basis of three types of registration error.

	Type 1 Error	Type 2 error		Type 3 error		Remaining radial bins
	Number of bins	Number of bins	Mean DSC and HD*	Number of bins	Mean DSC and HD*	
CEA1	24	8	DSC: 0.78 vs. 0.83** HD: 1.06 vs. 0.65	5	DSC: 0.79 vs. 0.90** HD: 1.01 vs. 0.59	73/96 (76.0%)
CEA2	13	11	DSC: 0.70 vs. 0.81** HD: 1.23 vs. 0.75	8	DSC: 0.84 vs. 0.89** HD: 0.87 vs. 0.55	60/88 (68.2%)

*DSC = Dice Similarity Coefficient, HD = Hausdorff Distance (mm)

**Mean DSC and HD values are given before vs. after exclusion of bins with error

In both CEAs, the majority of exclusions of radial bins were due to type 1 errors (37 of 184 bins), representing histological artefacts. Errors of type 2 and 3, representing registration mismatches between imaging modalities were found in a small number of bins: 19 of 184 bins had a type 2 error and 13 of 184 bins were found to have a type 3 error. Excluding these bins, based on consensus between three observers, improved the mean DSC and HD values for both CEAs (Table 1). The average DSC_type2 became 0.82 for both CEAs. The average DSC_type3 became 0.90. The average HD for both CEAs was 0.70 mm after exclusion of bins on basis of type 2 errors. After exclusion of type 3 error-containing bins, average HD reduced to 0.57 mm.

### Simulation of image registration mismatch

**Fig 5:**
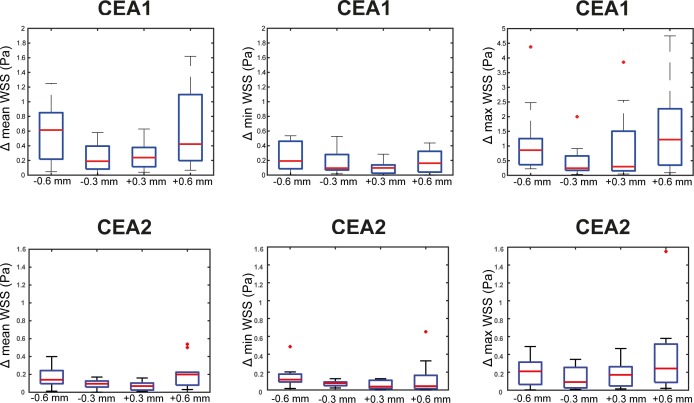
Average absolute variation in mean, minimum and maximum WSS values for axial locations after relocation of WSS data in CEA1 and CEA2. Delta represents difference in WSS value compared to original registration. Note that the Y-axis scaling differs in the Δmax WSS boxplot.

The WSS data was relocated by -0.6 mm, -0.3 mm, +0.3 mm and +0.6 mm (Fig 3B) in z-direction, with respect to the stack of histology images. For each registration case, the mean WSS value per radial histology bin was calculated. For each axial location, the WSS values of the radial bins were averaged. In Fig 5, the distribution of WSS values over axial locations for each case of relocation of the WSS data is visualised for CEA1 and CEA2 and compared to the original registration. The deviation between WSS values in the original registration and WSS values in cases of simulated registration mismatch increased with increasing length of axial mismatch. Combining the results of both CEAs, mean WSS varied on average 0.25 Pa in case of 0.3 mm relocation and 0.59 Pa in case of 0.6 mm relocation. In none of the cases the difference in mean, minimum or maximum WSS after relocation, with respect to the WSS values of the original registration, were significant.

**Fig 6:**
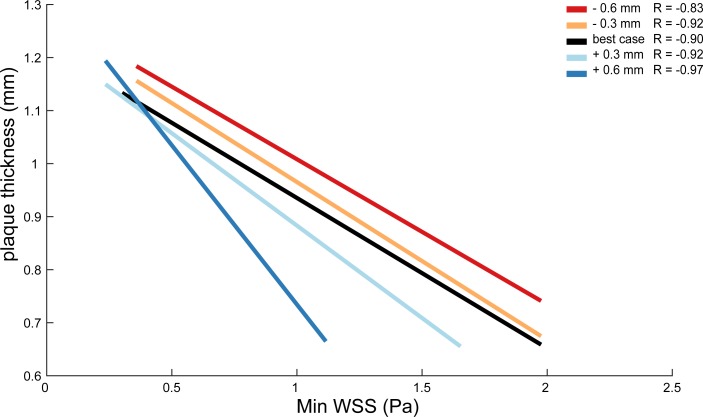
Correlation between minimal WSS and plaque thickness for one axial location. Variation in minimum WSS-plaque thickness correlation, resulting from relocation of the WSS data in z-direction, is shown.

We tested the effect of relocation of the WSS data on the correlation between WSS and plaque thickness. Per axial location, we plotted, for each radial bin, the mean, minimum and maximum WSS against the average plaque thickness and calculated the R value of the original registration. In two axial locations, the correlation between minimal WSS and plaque thickness was negative and significant. In all other axial locations, no significant correlation between plaque thickness and mean, minimum or maximum WSS was found. Considering the negative correlation between WSS and plaque thickness found in one axial location: this correlation remained significant in all cases of 0.3 mm relocation and in two out of four cases of 0.6 mm relocation. Fig 6 shows the results of this analysis for one axial location. After relocating the WSS data, the R value of the original registration (R = -0.90) increased to R = -0.97 after application of a +0.6 mm relocation of the WSS stack (Fig 6). The R value decreased to R = -0.83 when a –0.6 mm relocation was applied (Fig 6). In this example, significance was lost only in case of -0.6 mm relocation of WSS data. A mismatch of 0.3 mm changed the value of the correlation coefficient between mean WSS and plaque thickness by 0.045 on average. A mismatch of 0.6 mm changed the correlation coefficient between mean WSS and plaque thickness by on average 0.049, compared to the original registration.

## Discussion

We developed the first MRI-based pipeline to register WSS data to histology images. This method enables patient-specific investigation of correlations between WSS and histology-based plaque composition. We demonstrated that a mismatch of one slice does not significantly affect WSS distribution or the relation between WSS and plaque thickness.

In terms of image registration, this pipeline is an improvement over a previous CT-based method developed by our group [21]. Our new pipeline is fully based on MRI, and therefore requires less image registration steps. Compared to CT, i*n vivo* MR imaging is less harmful to the patient as it does not involve ionising radiation. In addition, patient-specific blood flow and WSS information can be derived from MRI scans [26]. Finally, MR imaging has the potential to image different plaque components [32], that can serve as additional landmarks for image registration, in addition to the lumen and vessel wall contours used in this pipeline.

In addition to our objective of registering WSS data to histology, the developed image registration method can also serve other MRI-based image registration purposes, such as comparison of plaque imaging sequences or validation of image segmentation algorithms.

We assessed registration accuracy and excluded radial bins on the basis of three error types. By this selection process, 28% of radial bins was excluded from the data-sets. Only a minor part of radial bins was excluded due to insufficient image registration, as represented by type 2 and type 3 errors. Reliable matching between histology and WSS was also evaluated by DSC and HD. After removing insufficiently matched radial bins from the data-sets, average HD values ranged from 0.57 mm to 0.70 mm. This distance between en face photo contours and histology or WSS contours was in the order of magnitude of the *in vivo* MRI resolution.

The applied image registration algorithms required user-defined matching of the bifurcation slices and segmentation of lumen and outer wall contours in different imaging modalities. However, image registration accuracy might be compromised for plaque samples with a relatively large axial length and/or a concentric plaque. In these cases, accuracy of the pipeline can be improved if rigid registration can reliably be based on multiple image slices. To achieve this, additional anatomical landmarks are needed, that are clearly recognisable in the imaging modalities to be registered. Large calcium spots might be good landmarks, as these can be imaged by a combination of MRI sequences [33] and remnants of large calcium areas are also visible in the en face photos and in the histological sections.

The rigid registration of *in vivo* and *ex vivo* MRI is based on the matching of a single image slice. A potential registration error of one image slice in this procedure would equal the MRI resolution in z-direction, i.e. 0.6 mm. After relocation of a half image slice with respect to the original registration, mean WSS values varied on average 0.25 Pa. Simulating the mismatch of a complete MRI slice resulted in an average deviation in mean WSS of 0.59 Pa. As expected, larger mismatches resulted in larger WSS deviations. Although a WSS value difference of 0.59 Pa appears substantial, deviations in WSS values in the same order of magnitude were found to result from variations in vessel segmentations for WSS calculations [34]. As we plan to use this method for assessing possible correlations between WSS and histological parameters, the effect of possible registration and segmentation inaccuracies on found correlations should be carefully documented.

The carotids included in this study are representative geometries, that have a stenosis degree of >70%. The nonsignificant change in WSS values after relocation suggests that the axial gradients in WSS data are not large enough to cause significant changes, even in case of mismatch of a complete MRI slice. Plaques with relatively irregular lumen outlines will show larger gradients in WSS data. In those cases, a registration mismatch is more likely to cause a significant change in WSS data on radial bins. In future applications of this pipeline, the 'smoothness' of the lumen should thus be carefully assessed and, in case of irregular plaques, the applicability of this image registration pipeline should be re-assessed.

We determined whether a registration mismatch would influence the correlations we want to investigate with the pipeline, namely the identification of a possible relation between WSS and a histology parameter. For this purpose, we analysed the correlation between WSS and plaque thickness. In two axial locations close to the bifurcation, we found a significant negative correlation (p<0.05) between minimum WSS and plaque thickness. The negative correlations remained significant in case of a 0.3 mm mismatch in WSS data. A 0.6 mm WSS relocation, resulting in a larger change in WSS, weakened the correlation to non-significance in two out of four cases. For all cases, the R value of the original registration however, remained negative. Non-significant R values in other axial locations in case of original image registration remained non-significant. Considering the correlation between mean WSS and plaque thickness, the R values after relocation deviated from the R values of the original registration by 0.045–0.048 on average. Similar to the WSS patterns, the correlation between WSS and plaque composition should be reassessed for plaques with a highly irregular lumen, potentially requiring additional registration landmarks.

In conclusion, our novel MRI-based pipeline can match histology to patient-specific WSS data. This method can now be used to investigate the relation between the hemodynamic environment and features of plaque vulnerability.
